# Current status of immunodeficient mouse models as substitutes to reduce cat and dog use in heartworm preclinical research

**DOI:** 10.12688/f1000research.149854.2

**Published:** 2024-09-11

**Authors:** Jessica L Dagley, Utami DiCosty, Crystal Fricks, Abdelmoneim Mansour, Scott McCall, John W McCall, Mark J Taylor, Joseph D Turner

**Affiliations:** 1 Department of Tropical Disease Biology, Centre for Drugs and Diagnostics, Liverpool School of Tropical Medicine, Liverpool, Merseyside, UK; 2 TRS Labs, Inc., Athens, GA, USA; 3 Department of Infectious Diseases, College of Veterinary Medicine, University of Georgia, Athens, GA, USA

**Keywords:** Dirofilariasis, heartworm, parasitology, anthelmintic, anti-parasitic drugs

## Abstract

Chemoprophylactic prevention of veterinary heartworm disease in companion animals, caused by the vector-borne nematode parasite *Dirofilaria immitis,* is a multi-billion-dollar global market. Experimental use of cats and dogs in preclinical heartworm drug testing is increasing due to evolving drug-resistance to frontline macrocyclic lactones and renewed investment in alternative preventative drug research. We and others recently published data demonstrating proof-of-concept of utilising lymphopenic severe-combined immunodeficient (SCID) or Recombination Activating Gene (RAG)2 deficient mice with additional knockout of the IL-2/7 receptor gamma chain (γc) as alternative preventative drug screening research models of dirofilariasis. Here we summarise the current knowledge of candidate immunodeficient mouse models tested, including a comparison of susceptibility using different background strains of mice, different *D. immitis* isolates, following use of anti-inflammatory treatments to further suppress residual innate immunity, and efficacies achieved against different reference anthelmintics. We supplement this precis with new data on treatment response to the veterinary anthelmintic, oxfendazole, and initial evaluation of *D. immitis* susceptibility in CB.17 SCID and C57BL/6 RAG2 ^-/-^γc ^-/-^ mice. We conclude that in addition to NSG and NXG mice, RAG2 ^-/-^γc ^-/-^ mice on either a BALB/c or C57BL/6 background offer an alternative screening model option, widening access to academic and commercial laboratories wishing to pursue initial rapid *in vivo* drug screening whilst avoiding potentially unnecessary cat or dog testing.

Research highlights **Scientific benefits(s):**

• A variety of immunodeficient mouse models of *Dirofilaria immitis* (heartworm) are reproducibly susceptible to tissue-phase L4 stage larvae.
• Oxfendazole’s effectiveness in reducing *D. immitis* tissue-phase larvae demonstrates potential use as a heartworm preventative.

**3Rs benefits(s):**

• As alternative *in vivo* models for heartworm, mice have the potential to reduce the overall use of specially protected species, cats and dogs, in heartworm preventative compound screening.
• Mice present with no clinical signs of tissue-phase *D. immitis* infection over 5 weeks, categorising this model as a ‘mild procedure’.
• Mouse models have the potential to be used as a screening model before moving onto more sentient and highly protected species, potentially reducing the number of chronic procedures by 67% and longitudinal infection studies risking moderate to severe welfare arising in cats and dogs.

**Practical benefits(s):**

• The use of rodent heartworm models has advantages in comparison to cats and dogs for preliminary drug screening such as ease of pharmacology standardisation, reduced costs of maintenance and higher throughput.
• Increased variety of commercially available mouse strains susceptible to heartworm extends global access for heartworm drug testing in laboratories where heartworm infectious larvae can be supplied.

**Current applications:**

• Evaluation of anti- *Wolbachia* compounds at our laboratory, as a novel approach to heartworm prevention.
• Adoption in industry labs for more widespread use as an initial *in vivo* screening model for preventative research and development.

**Potential applications:**

• Onward use of models in other basic and applied biological research e.g. heartworm developmental biology, mechanisms of drug resistance, drug repurposing, immune-mediated control of heartworm including vaccine research and biomarker discovery.

## Introduction

Affecting felids and canids, heartworm disease is caused by the mosquito-borne filarial nematode, *Dirofilaria immitis.* With vectors including the invasive *Aedes albopictus,* heartworm has an emerging global distribution ( Simón *et al.*, 2012; Noack *et al.*, 2021; Morchón *et al.*, 2022). Canine chronic-progressive heartworm disease can result in heart failure following establishment of adult worms within the pulmonary vascular system. In cats, immature worms can result in potentially lethal heartworm-associated respiratory disease ( McCall *et al.*, 2008). Humans are at risk of developing abbreviated zoonotic infections, with increasing reported incidence ( Reddy, 2013). Human pulmonary lesions formed by infections are frequently confused with tumours ( Saha *et al.*, 2022). A related subcutaneous parasite, *D. repens,* is also widespread in Europe and Asia, risking renal damage in dogs and zoonotic ocular-dermal pathologies ( Genchi and Kramer, 2017; Noack *et al.*, 2021).

Drugs available for safe prevention and post-diagnosis treatment of heartworm disease are limited. The arsenical injectable, melarsomine, is the only registered cure for adult heartworm ( Self *et al.*, 2019; Morchón *et al.*, 2022). Melarsomine is not registered for use in cats and risks severe adverse events in dogs, requiring complex protracted case management, exercise restriction and supplementary treatments. Comparatively, primary control of heartworm relies on chemoprophylaxis using macrocyclic lactones (ML). Despite high efficacy of MLs during the first 60 days of *D. immitis* infection, concerns have been raised regarding the development of resistant isolates following their widespread utilisation within veterinary medicine. Resistance of *D. immitis* has been formally demonstrated within both field and laboratory settings, with “JYD-34” and “ZoeLA” isolates identified as ivermectin-resistant by laboratory-based validation ( McTier *et al.*, 2019; Prichard and Geary, 2019). Thus, there is a growing need for new heartworm chemoprophylactic drugs utilising a novel mode of action ( Turner *et al.*, 2020).

Until recently, only laboratory-reared cats and dogs have been validated for *in vivo* drug screening of preventative heartworm drug compounds following experimental infections. Ethical concerns arise following the use of such highly sentient animals, categorised with non-human primates as specially protected species under UK law. Additionally, to satisfy regulatory requirements that new prophylactic formulations prevent arrival of adult worms in the heart and lungs, studies are necessarily lengthy (≥6 months) and are vulnerable to moderate to severe complications. This is particularly evident in experimental cat infections due to potentially lethal immune-pathological respiratory disease when immature worms die in the lungs ( Dillon *et al.*, 2017). Finally, using cats and dogs faces practical challenges of keeping large laboratory-bred animals for long time-periods and limits throughput for drug development. Our analysis of published experimental heartworm studies between 2015-2020 identified that 1324 lab-reared cats and dogs have been documented in heartworm experimental research (221 per annum) with the majority (63%) used in drug testing.

Following previous success by our laboratory and others in developing rodent models for the medically important filarial nematodes *Brugia malayi, Onchocerca volvulus,* and *Loa loa* ( Halliday *et al.*, 2014; Pionnier *et al.*, 2019; Marriott *et al.*, 2022), investigation into the permissiveness of mice to *D. immitis* has recently been demonstrated by our laboratories ( Marriott *et al.*, 2023) and independently by Hess *et al.* (2023).

Here we summarise the status of heartworm immunodeficient mouse models in terms of *D. immitis* isolates, strains of inbred mutant and genetically modified mice, infection durations, and validations of drug testing models in terms of different anthelmintic efficacies. We supplement prior data with new findings demonstrating Georgia III strain *D. immitis* recoveries from NSG mice do not significantly vary at 5 weeks in comparison to earlier time points, and further validate a five-week drug screen, with single daily drug exposures at 4-week intervals using a novel reference veterinary filaricide, oxfendazole. We report evaluations of two additional commercially available mouse strains, C57BL/6NTac.Cg- *Rag2 ^tm1Fwa^ Il2rg ^tm1Wjl^* (RAG2 ^-/-^γc ^-/-^), and C.B- *Igh-1 ^b^*/IcrTac- *Prkdc ^scid^* (C.B-17 SCID) with or without additional steroid treatment, for susceptibility to *D. immitis* tissue-phase larval infection.

## Methods

### Animals

Male NOD.Cg- *Prkdc ^scid^ Il2rg ^tm1Wjl^*/SzJ (NSG) mice were purchased from Jax Labs, USA. Male C57BL/6NTac.Cg- *Rag2 ^tm1Fwa^ Il2rg ^tm1Wjl^* (B.6 RAG2 ^-/-^/γc ^-/-^) and C.B- *Igh-1 ^b^*/IcrTac- *Prkdc ^scid^* (C.B-17 SCID) mice were purchased from Taconic, USA. Mice were 5-7 weeks old and 20-30g at the start of study. All mice were group housed at TRS Labs and allowed minimum seven days acclimation before study, kept in stacked cages and with access to food and water *ad libitum.*

### *Dirofilaria immitis* L3 production

*Dirofilaria immitis* Georgia III (GAIII) isolate microfilariae in dog blood were fed to *Aedes aegypti* female mosquitoes (Liverpool strain) using a glass feeder at a density of 1,000-2,500 mf/ml with third-stage infective larvae (L3) collected 14 days later following protocols by Marriott *et al.* (2023). Mosquitoes were kept at temperatures 75-80 ^o^F, humidity at 72-95%.

### Animal infections and dosing

**Figure 1.**
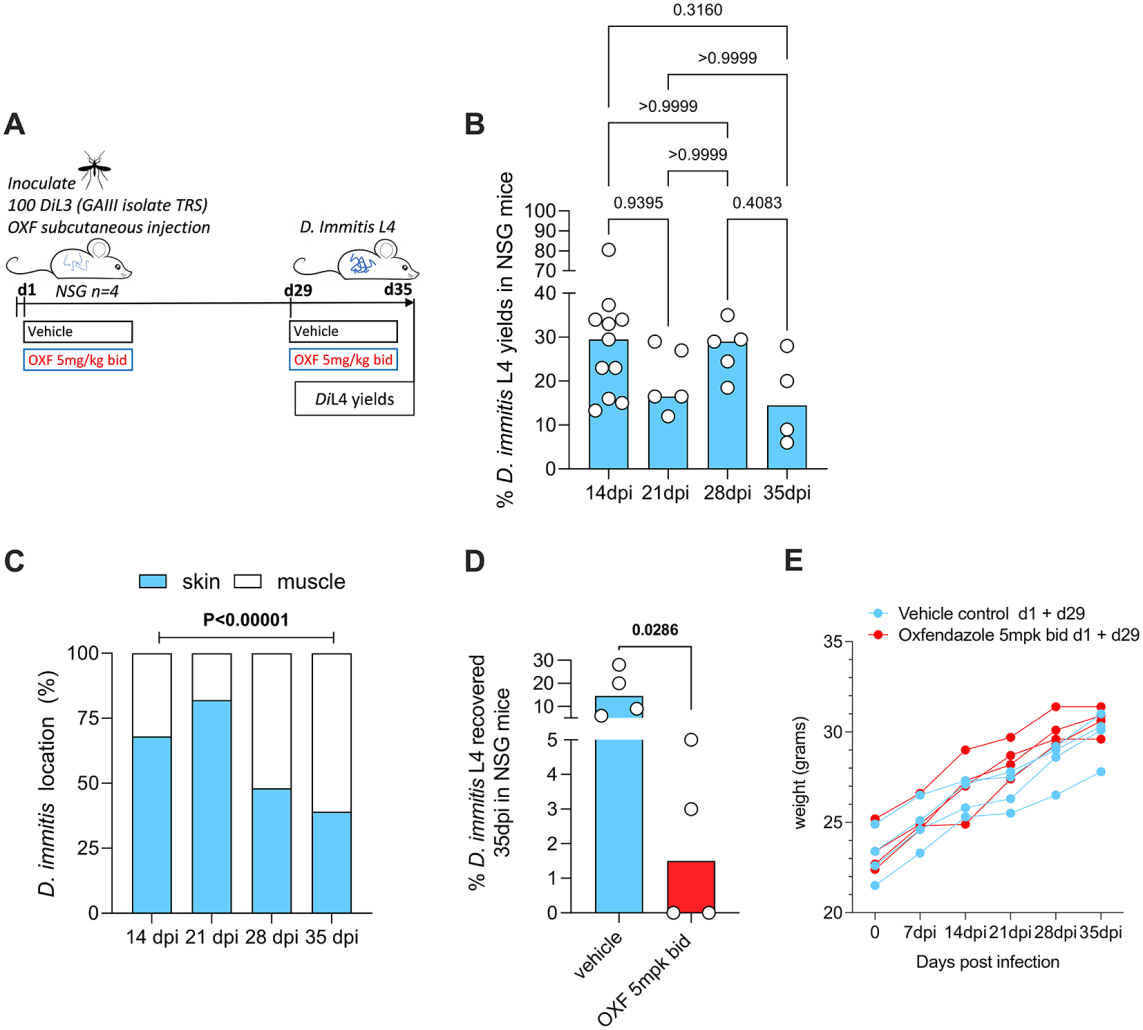
Schematic of experimental design (A). *D. immitis* L4 recovered from NSG mice 2-5 weeks post infection, *Di*L3 expressed as % of initial inoculate (B). Average proportions of L4 larvae in skin/subcutaneous tissue vs muscle (C). Recoveries following oral dosing with bi-daily (bid) oxfendazole (d1+d29) (5 mg/kg), or vehicle control (D). Weight changes in individual mice (E). Bars are median recoveries (B,D) or mean proportions (C) from a single experimental group of 4-5 mice or two-independent experiments combined (2 week data) with 2-to-4-week data previously published by Marriott *et al.* (2023). Significant differences determined by Kruskal–Wallis one-way ANOVA with Dunn’s multiple comparison’s tests except (C) where the difference in proportions was tested by 2x4 Chi-Square test for trend. Significant differences (P≤0.05) are indicated in bold, no data excluded.

**Figure 2.**
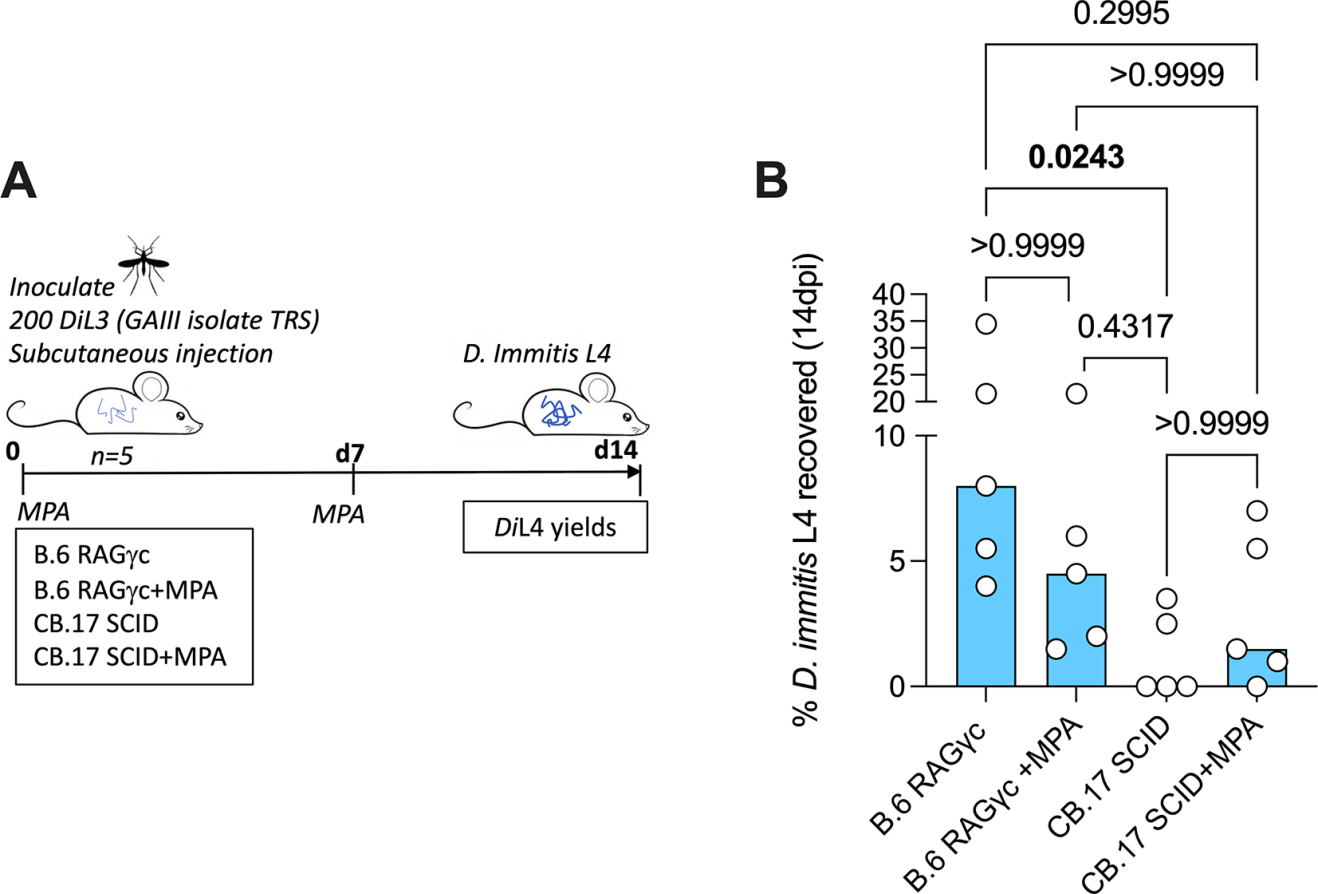
Schematic of experimental design (A). *D. immitis* L4 recovered from B.6 RAG2 ^-/-^/γc ^-/-^ and CB.17 SCID (+/- MPA treatment) 2 weeks post-infection with 200 GAIII *Di*L3 expressed as % of initial inoculate ( **B**). Bars are median recoveries from a single experimental group of 5 mice. Significant differences determined by Kruskal–Wallis one-way ANOVA with Dunn’s multiple comparison’s tests. Significant differences (P≤0.05) are indicated in bold, no data excluded.

NSG mice ( Figure 1A) were subcutaneously inoculated with 100 GAIII *Di*L3 into the flank and maintained until 5 weeks post-infection. Animals were allocated to treatment groups via cage (non-randomised), due to logistical constraints, experimental unit being a single animal. Treatment group (n=4 mice) received oral 5mg/kg bi-daily dose of oxfendazole (d1+d29). Oxfendazole was suspended in standard suspension vehicle (SSV; 0.5% carboxymethyl cellulose, 0.5% benzyl alcohol, 0.4% tween 80, 0.9% NaCl). B.6 RAG2 ^-/-^/γc ^-/-^ (n=5 mice) and C.B-17 SCID (n=5 mice) ( Figure 2A) received a subcutaneous injection of 2mg methylprednisolone acetate (MPA) in 200uL ddH _2_O. Control groups received a matching volume of ddH _2_O. Dosing was immediately followed by subcutaneous inoculation of 200 GAIII *Di*L3 into the flank. MPA dosing was repeated on d7, and mice sustained until 14 days post-infection (no blinding used during inoculation or dosing). All mice were monitored daily for welfare and weighed weekly, no animals excluded during the study. Those monitoring and weighing animals were aware of group allocation.

### Parasite collection

Mice were humanely euthanised by schedule one (rising CO _2_) two or five weeks post-infection, dependent on study design ( Figure 1A; Figure 2A). Collection and visual quantification of fourth-stage larvae (L4) using a light microscope followed protocols by Marriott *et al.* (2023).

### Statistical analysis

For deriving group size for drug testing, we utilised data of average yield and variation of GAIII larvae at 4 weeks post-infection in NSG mice (27.3±6.2, n=5) ( Marriott *et al.*, 2023) to calculate effect size and statistical power of minimum 70% reduction by drug treatment (e.g. predicted mean number of L4 larvae=8.2±1.9, d=4.2, power>0.9, n=3 per group, 2-tailed independent T test, alpha=0.05, calculated in G*Power 3.1). We included an additional mouse per group as mitigation in case of early welfare issues.

Tests were performed using GraphPad Prism 9.1.2. D’Agostino and Pearson omnibus Shapiro-Wilk normality testing indicated non-parametric analyses. Mann-Whitney tests or Kruskal Wallis with Dunn’s *post hoc* tests were used to compare quantitative differences. Chi-square tests for trend were used to assess categorical data over time. Statistical significance was defined as P≤0.05, experimental unit being a single animal. Group allocation was not specified to those conducting data analysis.

## Results

### GAIII *D. immitis* persist for five weeks and are susceptible to oxfendazole in NSG mice

Our laboratories previously demonstrated viable L4 larval yields two-to-four weeks post-infection using NSG or NXG mouse strains, following infection with Missouri (MO) or GAIII *D. immitis* ( Table 1) ( Marriott *et al.*, 2023). Hess *et al.* (2023) also demonstrated permissiveness of NSG mice to the MO isolate and the ML-resistant isolate, JYD-34, extending evaluations up to six weeks (summarised in Table 1). We therefore investigated the ability of NSG mice to sustain GAIII isolate infections for five weeks ( Figure 1A). Following subcutaneous inoculations of 100 *Di*L3, we reproducibly recovered GAIII *D. immitis* L4 on d35 dpi (4/4), median recovery rate of 14.5% (range 6-26%). This was not significantly variable when compared with GAIII L4 yields priorly attained at 2-4 weeks post-infection by Marriott *et al.* (2023) ( Figure 1B). At five-weeks, the majority (61%) of larvae were recovered from muscle tissues ( Figure 1C). Comparing to prior data at 14, 21 and 28-days post-infection, a significant linear increase in GAIII *D. immitis* developing larvae migration into muscle tissues over time was apparent (chi-square test for trend, 30.3, P<0.0001, Figure 1C). With the advantage that the impact of two daily chemoprophylactic exposures spaced 4 weeks apart could be evaluated within this extended timeframe in future studies (emulating monthly oral exposures in cats and dogs), we tested efficacy of a 5 mg/kg bid oral regimen of the benzimidazole oxfendazole, selected based on recent evidence it can mediate curative efficacies after short-course exposures in a filariasis infection model ( Hubner *et al.*, 2020). After two exposure cycles (d1+d29), oxfendazole mediated a median 90% reduction in *D. immitis* L4 compared with controls (d35), curing two out of four mice ( Figure 1D). Efficacy was comparable to reductions in larvae in MO, GAIII and JYD isolates treated with macrocyclic lactone regimens in NSG mice (data summarised in Table 2). Over the 35-day infection course, mice displayed no adverse behavioural changes determined during daily anecdotal observations by a veterinarian, and gained weight ( Figure 1E), indicating infections and drug dosing did not cause overt clinical welfare signs.

**Table 1.** Summary of *D. immitis* isolates and mouse strains tested as suitable tissue-phase canine heartworm larval infection models.

Model background strain (Supplier)	Maximum time post-infection evaluated yield (Median % inoculate recovered) (Range, n) *				
	MO *Di*L3 (LSTM UK)	MO *Di*L3 (UKB DE)	GAIII *Di*L3 (TRS USA)	MO *Di*L3 (TJU USA)	JYD-34 *Di*L3 (TJU USA)
Wild-type (C57BL/6J) ^1^ B.6 (Charles River, Jackson Labs)				42dpi 0% (0-0, n=5)	
Wild-type (NOD/ShiLt) ^1^ NOD (Jackson Labs)				42dpi 0% (0-0, n=5)	
SCID (NOD.Cg-Prkdcscid) ^1^ NOD (Jackson Labs)				42dpi 0% (0-0, n=5)	
NSG (NOD.Cg- *Prkdc ^scid^ Il2rg ^tm1Wjl^*/SzJ) ^1^^,^^2^^,^^3^ NOD (Charles River, Jackson Labs)	14dpi 5% (2-24, n=21)		35dpi 15% (6-28, n=5)	42dpi ^3^ 30% (1-25, n=35)	42dpi ^3^ 28% (8-30), n=21)
NXG (NOD- *Prkdc ^scid^-IL2rg ^Tm1^*/Rj) ^2^ NOD (Janvier)	14dpi 6% (1-23, n=18)				
SCID (C.B- *Igh-1 ^b^*/IcrTac- *Prkdc ^scid^*) ^4^ CB.17 (Charles River, Taconic)			14dpi 1.5% (0-7, n=5)		
SCID (+MPA) ^4^ CB.17 (Charles River, Taconic)			14dpi 0% (0-4, n=5)		
SCID (B6.Cg-PrKdcscid/SzJ) ^1^ B.6 (Jackson Labs)				42dpi 0% (0-0, n=5)	
RAG2γc (C;129S4- *Rag2 ^tm1.1Flv^Il2rg ^tm1.1Flv^*/J) ^2^ BALB/c (Charles River, Jackson Labs)	14dpi 1.5% (0-3, n=5)				
RAG2γc (+MPA) ^2^ BALB/c (Charles River, Jackson Labs)	14dpi 6% (4-14, n=5)				
RAG2γc (C57BL/6NTac.Cg- *Rag2 ^tm1Fwa^ IL2rg ^tm1Wji^*/Rj) ^4^^,^^5^ B.6 (Taconic, Janvier)		180dpi 2% (0-8, n=4)	14dpi 8% (4-35, n=5)		
RAG2γc (+MPA) ^4^ B.6 (Taconic, Janvier)			14dpi 5% (2-22, n=5)		

^1^ Data from Hess *et al.* (2023). Data quantified from graphical representations.

^2^ Data from Marriott *et al.* (2023).

^3^ Susceptibility reported by Hess *et al.* (2023) up to 15 weeks, but no quantitative data is available after 42dpi.

^4^ TRS USA previously unpublished data.

^5^ Data from Risch *et al*. (2024). Data quantified from graphical representations. Data shown as ‘180dpi’, however nematodes may have been collected from 161-180dpi.

* Maximum time post-infection evaluated yield shown except for Risch *et al*. (2024) during which two later time points were also investigated (181-200dpi and 201-210dpi) from which no nematodes were recovered.

**Table 2.** Efficacy of anthelmintic regimens against *D. immitis* isolates in tissue-phase heartworm larval infection models.

Drug	Dose model	Regimen ^1^	Efficacy (n) time post-infection evaluated			
			MO *Di*L3 (LSTM UK)	GAIII *Di*L3 (TRS USA)	MO *Di*L3 (TJU USA)	JYD-34 *Di*L3 (TJU USA)
Ivermectin ^2^	0.005 mg/kg NSG	po qdx3 (d1/15/30)			57% (19) d42	
	0.01 mg/kg NSG	po qdx3 (d1/15/30)			73-76% (26) d42	30% (13) d42
	0.3 mg/kg NSG	po qdx3 (d1/15/30)			87% (21) d42	
	0.5 mg/kg NSG	po qdx3 (d1/15/30)			87% (18) d42	
	1.0 mg/kg NSG	po qdx3 (d1/15/30)			87% (11) d42	
	3.0 mg/kg NSG	po qdx3 (d1/15/30)			87% (6) d42	
Moxidectin ^2, 3^	0.01 mg/kg NSG	po qdx3 (d1/15/30)			96% (8) d42	88% (8) d42
	2.5 mg/kg NSG	sc qdx1 (d1)	65-80% (3) d14	60, 73, 75% (5) d14,21,28		
	2.5 mg/kg NXG	sc qdx1 (d1)	67-88% (5) d14			
Oxfendazole ^4^	5 mg/kg NSG	po bidx2 (d1/29)		90% (4) d35		

^1^ po = per oral, sc = subcutaneously, qd = once per day, bid = twice per day.

^2^ Mean efficacy compared with vehicle control, reported in Hess *et al.* (2023).

^3^ Median efficacy compared with vehicle control, reported in Marriott *et al.* (2023).

^4^ Median efficacy compared with vehicle, previously unpublished data.

### B.6 RAG2 ^-/-^/γc ^-/-^ and CB.17 SCID mice are susceptible to GAIII *D. immitis*

Hess *et al.* (2023) described mouse susceptibility to MO isolate *D. immitis* as specific to the NSG line, as other strains including lymphopenic SCID mice on NOD or B.6 backgrounds were refractory to infection (summarised in Table 1). NOD mice have inherent strain-specific deficiencies in the complement system ( Verma *et al.*, 2017) and thus combinations of these, or other background strain-specific immune gene mutations, combined with susceptibility of the introduced SCID mutation and IL-2Rγ ablation, may culminate in multiple immune-impairments sufficient to allow *D. immitis* survival and growth *.* However, in our prior study ( Marriott *et al.*, 2023), we identified compound lymphopenic (RAG2 ^-/-^) and IL-2Rγ deficiencies on a BALB/c background as susceptible to the *D. immitis* MO isolate at two weeks, with methyl-prednisolone (MPA) steroid treatment augmenting larval recoveries. We therefore examined two commercially accessible, alternative lymphopenic mouse strains on distinct genetic backgrounds: B.6 RAG2 ^-/-^/γc ^-/-^ and CB.17 SCID (BALB/c congenic), evaluating *D. immitis* GAIII L4 larval recoveries at 14dpi in groups of five mice with or without MPA treatment ( Figure 2A). Whilst all (5/5) B.6 RAG2 ^-/-^/γc ^-/-^ mice had recoverable *D. immitis* L4 larvae two weeks post-infection, only 2/5 CB.17 SCID mice were infection positive ( Figure 2B). Yields were significantly higher in RAG2 ^-/-^/γc ^-/-^ mice (median=8%, range=4-35% vs median=0%, range 0-4%, Kruskal Wallis One-Way ANOVA P=0.029, Dunn’s post-hoc test P=0.024). In B.6 RAG2 ^-/-^/γc ^-/-^ mice, MPA treatment did not significantly bolster yields (median 5%, range 2-22%). MPA treatment did increase the frequency of animals with infection in 4/5 CB.17 SCID mice, although heartworm larval recoveries were low and not significantly different to non-treated animals (median recovery=1.5%, range =0-7%) ( Figure 1B). Thus, we summarise that RAG2 ^-/-^/γc ^-/-^ mice are initially validated as an alternative susceptible tissue phase larval heartworm model, without requirement for steroid suppression of residual innate immunity. We summarise all mouse strains tested for *D. immitis* susceptibility in Table 1.

## Discussion

Following the success of Marriott *et al.* (2023) and Hess *et al.* (2023) in establishing a validated *D. immitis* immunodeficient NSG/NXG mouse preventative drug screening model, our current study demonstrates the ability of NSG mice to sustain GAIII *D. immitis* infection for up to five weeks post-infection with further evidence of larval migration from the skin and subcutaneous tissues into deeper musculature. This suggests that for the first 35 days, development of heartworm larvae emulates that of within natural hosts, whereby the L4-stage migrates from the subcutaneous space into muscles, penetrates the vasculature and arrives in the heart and lungs after 65-70 days ( Orihel, 1961; Supakorndej, McCall and Jun, 1994). Similarities in larval length at 14-35 days post infection in NSG mice: (1.5–1.8 mm, 14d & 3.5-4.0 mm, 35d) Hess *et al.* (2023) and NSG/NXG mice (1.2 – 2.8 mm, 14d) Marriott *et al.* (2023) are aligned with growth of *D. immitis* L4 in dogs during this timeframe (1.7–2.2 mm, 14d, 3.1-5.6 mm, 30d) ( Orihel, 1961; Lichtenfels, Pilitt and Wergin, 1987). Hess and colleagues identified that after 35 days, development of L4 in NSG mice diverged, with retarded growth in murine tissues. Hess *et al.* (2023) also observed no entry of larvae into the heart up to 15 weeks in NSG mice, suggesting L4 larvae may become arrested in development in subcutaneous tissues and muscle after 35 days infection. With the recent data of Risch *et al* (2024) demonstrating MO isolate adult development in B.6 RAG2 ^-/-^/γc ^-/-^ mice, arrested development of late-L4 appears specific to NSG mice, rather than a universal barrier to development in all immunodeficient mice. Regardless of long-term susceptibility, the window of aligned growth in NSG mice is encouragingly sufficient to allow testing of new preventative candidates in this model, utilising regimens emulating once-per-month exposures demanded by current target candidate drug profiles. In prior studies, single-dose injected moxidectin or 3x fortnightly oral ivermectin/moxidectin have been utilised for initial validations (summarised in Table 2). With no regimen demonstrating 100% effectiveness, as seen in dogs, this may indicate an ancillary requirement for host adaptive immune responses to deliver optimum ML efficacy, as prior discussed ( Marriot *et al*., 2023). When we tested oxfendazole in a daily exposure cycle spaced four weeks apart, we demonstrated 90% efficacy, extending validation of the NSG mouse model and demonstrating feasibility of once-per-month drug testing. We selected oxfendazole based on its registered use in companion animals, activity against L3 filarial larvae ( Jawahar *et al.*, 2021) and recent demonstrable curative activity against *Litomosoides sigmondontis* infection models ( Hubner *et al.*, 2020; Jawahar *et al.*, 2021). Oxfendazole may thus have the potential to be used as an alternative or in combination with ivermectin for monthly oral prevention of infection with drug-resistant *D. immitis* and should be scrutinised for dose-dependent efficacy against resistant isolates.

We explored additional laboratory inbred strains of mice and effects of MPA treatment. C57BL/6J, NOD (NOD/ShiLt), B.6 SCID, and NOD.SCID mouse strains previously investigated by Hess *et al.* (2023) were determined refractory to MO isolate *D. immitis* (summarised in Table 1). Here we identify C.B-17 SCID and B.6 RAG2 ^-/-^/γc ^-/-^ mice as susceptible to *D. immitis* GAIII isolate survival and growth over 14-days. MPA treatments were not necessary for susceptibility in B.6 RAG2 ^-/-^/γc ^-/-^ mice, simplifying onward use for drug testing and avoiding potential drug-drug interactions. MPA treatments were successful in increasing the infection success of CB.17 SCID mice, indicating that inherent immune traits varying between these different genetic backgrounds combined with lymphopenia and deficiency in IL-2/7 receptor signalling dictates early immune control of *D. immitis* larvae in mice. We and others have established both innate (natural killer cell, alternatively activated tissue resident macrophage) and adaptive (IL-4/5/13 producing CD4 ^+^ T cell) immune responses combine to orchestrate eosinophil-dependent immune response to developing *B. malayi* larvae in mice ( Turner *et al.*, 2018; Pionnier *et al.*, 2020, 2022). Additionally, while this report was under review, Risch *et al*. (2024) published their evaluation of B.6 RAG2 ^-/-^/γc ^-/-^ mice, demonstrating migration and long-term development of adult nematodes within the heart and lung vasculature (summarised in Table 1). However, some mice developed severe caval syndrome during these later stages of infection, and from a welfare aspect, it would be advisable to limit drug screening endpoints to the late-L4 tissue stage of infection which we have evaluated as a mild procedure. The variety of biological, pharmacological, and genetic modification tools in laboratory mice (many of which available on BALB/c or B.6 inbred backgrounds) may now be applied to pinpoint the basis of immunity against dog heartworm, potentially supporting rational vaccine design. Despite the presence of a patent pending or awarded, in Europe and other territories, (abandoned in USA), for use of mouse models, claims are restricted to the NSG mouse model applied to prophylactic anthelmintic drug screening ( Abraham *et al*., 2028). We therefore suggest that establishment of susceptibility in a variety of alternative mouse models summarised here will allow for unfettered access by the parasitology community for both basic and translational research.

One potential limitation of this new data is that power calculations (n=3) assumed a normal distribution whereas parasite yields were aggregated, requiring non-parametric testing. Despite a potential underpower, due to the potency of oxfendazole being >70% efficacy, and our inclusion of an extra animal per group, this did not affect determining a significant outcome. In future studies, researchers should be wary of aggregated distributions when determining sample size.

Current regulatory requirements demand 100% prophylactic activity in cats or dogs for registration of new heartworm preventatives, meaning it is not currently possible to completely avoid experimental use of cats and dogs. However, with a variety of susceptible mouse strains available (summarised in Table 1), some without current commercial use restrictions, we envisage these new models may become widely adopted by both academic, not-for-profit, and commercial organisations to produce L4 for *in vitro* drug titration evaluations and for initial triaging of compounds and exposure-regimen selections *in vivo.* Future adoption of immunodeficient mice as an initial frontline screen, with short timeframes and without notable impacts on weight or welfare changes arising during the tissue-phase infection period, has the potential to reduce the overall use-requirement of cats and dogs in experimental heartworm research by at least 50%.

## Ethics statement

Male NSG, CB.17 SCID and C57BL/6 RAG2 ^-/-^γc ^-/-^ mice were group housed at TRS Labs. within filter-top cages. Animals had continuous access to fresh sterile food, water, and enrichment throughout experiments. Weight was monitored weekly and welfare behaviour monitored daily. Humane endpoints were defined as >20% weight loss and/or observation of adverse behavioural changes which did not improve over a 6h observation period following any remedial treatment by study veterinarian including but not limited to: loss of mobility, starring coat, eye squint, pinched nose, ears pulled back, and/or laboured breathing. Studies were conducted in the USA and approved by the TRS Institutional Animal Care and Use Committee. Protocols were identical to prior approved studies conducted in the UK, approved in the UK by LSTM & University of Liverpool Animal Welfare and Ethics Review Boards and licensed by The UK Home Office Animals in Science Regulation Unit. The manuscript was written in adherence with the ARRIVE 2.0 guidelines.

## Data Availability

Figshare: Current status of immunodeficient mouse models as substitutes to reduce cat and dog use in heartworm preclinical research, https://doi.org/10.6084/m9.figshare.25250101.v1 ( Dagley *et al.*, 2024). This project contains the following underlying data:
- Supplementary data file_NC3Rs.xlsx Figshare: ARRIVE checklist, https://doi.org/10.6084/m9.figshare.25690062.v1 ( Dagley, 2024).
- ARRIVE NC3Rs_.pdf. Data are available under the terms of the Creative Commons Attribution 4.0 International license (CC-BY 4.0).
