# Supplementary material for: Current status of immunodeficient mouse models as substitutes to reduce cat and dog use in heartworm preclinical research
Source: F1000Res. 2024 Sep 11;13:484. Originally published 2024 May 17. [Version 2] doi: 10.12688/f1000research.149854.2 (PMC11259772; doi:10.12688/f1000research.149854.2)
Supplement: Peer review history

# Reviewer report: Reviewer response for version 1

**DOI:** 10.5256/f1000research.164362.r299372

**Published:** 2024-08-05

**Authors:** Jan Šlapeta

**Affiliations:** 1 The University of Sydney, New South Wales, Australia

Authors demonstrate recovery of *D. immitis *larvae from B.6 RAG2-/-/γc-/- mice without the need for methylprednisolone, thus simplifying onward use of mice for drug testing against *D. immitis *(Di).

 Oxfendazole is poorly soluble, would authors have data on Tmax and elimination halflife in mice and potentially in dogs? Generally, I would expect it will be rather quick as far as Tmax and half-life will be likely <12h. This information should be considered when discussing your results and the reality of oxfendazole use in dogs. I assume it was because you chosen "bid" dose to counter the rapid Tmax and elimination. In this context it would have been beneficial to measure the concentration and bioavailability at some key timepoints post dosing. The regimen chose with Di infection got me thinking why was infection at time point zero (day 1) chosen? More realistic dosing would have been only at 28-30 dpi, thus considering all the <30 days larvae.

 Could you elaborate on reasoning to increase to 200 Di L3 in some of your experiment? Would 200 L3 be more optimal for future experiments in general to increase the number of detectable larvae at the end of the experiment / power analysis?

 Fig 1D / same for Fig 2 B – I don’t think the % should be there, you're reporting real values and an arithmetic mean? Please check. It maybe or not maybe % but depends on the starting # either 100 or 200 L3 inoculated.

 I am not sure how you calculated your >90% +reduction (results section first paragraph). Please provide the calculation and values used.

 Authors should reconsider the introductory sentence (and later in the 3 ^rd^ paragraph); potentially misleading if they start with felids/cats. It is canids that are the principal and natural vertebrate/mammal hosts esp. domestic dog in this context where the disease and suffering is pronounced and requires intervention. Felids are in host situation just an aberrant host as with rare development of sexually mature L1 producing Di. I am personally not aware of the term “abbreviated” zoonotic infection. I fully acknowledge the presence of coin lesions in humans, but it is potentially misleading to focus on humans and so zoonosis when the real suffering and almost solely burden in dogs.

 In the second paragraphs authors note 60-days, I personally would be extremely cautious about using such number. The registration, most if not all, claim 28/30 days. The research reports cannot be considered as claims and so if any veterinarian would recommend 60 days it would be solely off-label use. I would exercise caution and suggest authors align with the registration claims. No need to go into detail re different MLs and if there is potential or not based on research articles.

Is the work clearly and accurately presented and does it cite the current literature?

Yes

If applicable, is the statistical analysis and its interpretation appropriate?

Yes

Are all the source data underlying the results available to ensure full reproducibility?

Yes

Is the study design appropriate and is the work technically sound?

Yes

Are the conclusions drawn adequately supported by the results?

Yes

Are sufficient details of methods and analysis provided to allow replication by others?

Yes

Reviewer Expertise:

Veterinary and molecular parasitologist

I confirm that I have read this submission and believe that I have an appropriate level of expertise to confirm that it is of an acceptable scientific standard.

# Reviewer report: Reviewer response for version 1

**DOI:** 10.5256/f1000research.164362.r299367

**Published:** 2024-07-24

**Authors:** Qian Han

**Affiliations:** 1 Hainan University, Haikou, Hainan, China

The use of mouse models in heartworm drug screening research holds great significance. It offers an ethical alternative to the experimental use of cats and dogs, reducing the reliance on these companion animals for preclinical drug testing and minimizing potential harm and discomfort to them. Mouse models provide a controlled environment that allows for precise manipulation and monitoring of variables. This enables researchers to systematically study the efficacy, mechanism of action, and potential side effects of different heartworm drugs in a more standardized and reproducible manner. Moreover, the development of mouse models helps in understanding the immune responses and pathophysiological processes underlying heartworm infection and drug treatment. This fundamental knowledge can guide the development of more effective and targeted therapeutics in the future. In conclusion, mouse models are a valuable tool in heartworm drug screening research, accelerating the drug development process and contributing to the fight against this important veterinary parasitic disease.

 The presented research provides a valuable contribution to the field of chemoprophylactic prevention of veterinary heartworm disease via the exploration of immunodeficient mouse models in drug screening. The comprehensive comparison of different mouse models, worm isolates, anti-inflammatory treatments, and anthelmintic offers a detailed analysis that strengthens the reliability and validity of the proposed screening models. The addition of new data on the treatment response to oxfendazole and the evaluation of susceptibility in specific mouse strains adds to the novelty and practical application of the study.

 However, according to the information of author's current and previous studies, most heartworm larvae are in the tissues, such as muscle and skin, instead of blood vessels. The differences of worm distributions, and drug distribution, metabolism, and excretion in different animals may lead differences in drug efficacy. Authors may consider pharmacokinetic evaluations in combination with drug screening for the interpretation of screening results.

Is the work clearly and accurately presented and does it cite the current literature?

Yes

If applicable, is the statistical analysis and its interpretation appropriate?

Yes

Are all the source data underlying the results available to ensure full reproducibility?

Yes

Is the study design appropriate and is the work technically sound?

Yes

Are the conclusions drawn adequately supported by the results?

Yes

Are sufficient details of methods and analysis provided to allow replication by others?

Yes

Reviewer Expertise:

veterinary parasitology, vector borne pathogens

I confirm that I have read this submission and believe that I have an appropriate level of expertise to confirm that it is of an acceptable scientific standard.

# Reviewer report: Reviewer response for version 1

**DOI:** 10.5256/f1000research.164362.r299364

**Published:** 2024-07-19

**Authors:** Timothy G Geary

**Affiliations:** 1 McGill University, Montreal, Québec, Canada

This is a valuable addition to the burgeoning literature in this area and addresses an important topic. It is well-written, clear and concise. I have a few points for the authors to consider.

 First, two additional recent reports perhaps should be cited. One( Mizuseki M, et al., 2024 [Ref-1]) is perhaps slightly less relevant, but the other (Risch F, et al., 2024 [Ref-2]) should be cited and discussed, as it reports the development of adult stages in mice. I understand that the field moves quickly, but the value of the current manuscript will be enhanced if these papers are included.

 Two, macrocyclic lactones were less potent and less efficacious in the mouse strains compared to dogs. These drugs are believed to enlist the host immune response in vivo, which obviously may be compromised in these host strains. Some additional discussion around this issue is warranted.

 Finally, I believe the authors should acknowledge that intellectual property has been filed on at least one of the models (and the claims may be sufficiently broad to cover the others). I believe that patent may have been granted in the EU (at least). This is relevant because it may block drug screening by others. I think it is essential for the authors to discuss this. In addition, a granted patent covers the use of immunosuppressed rats as hosts for this parasite. I believe patents are citable, and this one may also cover ore broadly the use of immunocompromised rodents for drug screening for heartworm preventatives (the authors should check). Interestingly, effects of macrocyclic lactones in these rates closely resemble those in dogs (potency and efficacy), an interesting difference from the mouse that really should be discussed in the current manuscript.

Is the work clearly and accurately presented and does it cite the current literature?

No

If applicable, is the statistical analysis and its interpretation appropriate?

Yes

Are all the source data underlying the results available to ensure full reproducibility?

Yes

Is the study design appropriate and is the work technically sound?

Yes

Are the conclusions drawn adequately supported by the results?

Yes

Are sufficient details of methods and analysis provided to allow replication by others?

Yes

Reviewer Expertise:

Anthelmintic pharmacology (basic, clinical, discovery, development, resistance); molecular basis for the host-parasite interaction

I confirm that I have read this submission and believe that I have an appropriate level of expertise to confirm that it is of an acceptable scientific standard.

## References

[1] : Development of a novel rodent model for dog heartworm microfilaremia using the severe-combined immunodeficiency mouse. *Sci Rep* .2024;14(1) : 10.1038/s41598-024-63165-x 13741 10.1038/s41598-024-63165-x 38877072 PMC11178764

[2] : Development of adult Dirofilaria immitis worms in the Rag2/Il-2rγ-/- mouse model. *Int J Parasitol* .2024;54(5) : 10.1016/j.ijpara.2024.01.004 195-200 10.1016/j.ijpara.2024.01.004 38246406

# Response

**Published:** 2024-07-29

**Authors:** Jessica Dagley

**Affiliations:** Tropical Disease Biology, Liverpool School of Tropical Medicine, Liverpool, Merseyside, UK

Thank you for your feedback and comments. We hope we have addressed your points in the updated version and have some comments below:

 - While this article was under review, the report by Risch F et al., 2024 was published. We agree this is an important article, which corroborates our data herein, and indicates independent verification of susceptibility to D. immitis isolates in B.6 RAG2-/-gc mice. Importantly this research also demonstrates that there is no biological barrier to the migration and development of adults within the heart and lung vasculature in mice. We have added to data and discussion highlighting this research within our article.

 - We believe the Mizuseki M et al., 2024 report to be less relevant to our report, as it details a microfilarial-specific infusion model, and thus do not incorporate into comparisons within this specific brief report.

 - While the discussion around the mode of action of MLs is warranted, we have discussed this in much greater detail within an earlier publication (Marriott et al., 2023). We have therefore referenced this, but due to this being a brief report, will not discuss in greater detail within the text.

 - We appreciate the point raised regarding the rat patent; however, we do not deem it appropriate to discuss data within patents filed where data is not yet peer reviewed and published.

 - Regarding the mouse patent filed by Boehringer Ingelheim and awarded in Europe (abandoned in USA) based on the work of Hess et al., 2023, we suggest the patent claims are restricted to the NSG model, and a number of emerging alternative immunodeficient mouse lines proven to be susceptible to D. immitis infection should be available for use without restriction in both academia and industry. We have added some discussion regarding this into the main body of text.

# Reviewer report: Reviewer response for version 1

**DOI:** 10.5256/f1000research.164362.r299366

**Published:** 2024-07-19

**Authors:** Constantin Constantinoiu

**Affiliations:** 1 James Cook University, Townsville City, Queensland, Australia

Current status of immunodeficient mouse models as substitutes to reduce cat and dog use in heartworm clinical research

 The paper summarises previous research on the use of mice as models of infection with *Dirofilaria immitis* and furthers extends the previous research by demonstrating that:

- GAIII isolates of *D. immitis* can develop up to 5 weeks in NSG mice and that they are susceptible to the action of Oxfendazole;
- GAIII isolate of *D. immitis* can develop up to 2 weeks in RAG2 mice with requirement for suppression of the residual immunity by MPA.

The paper is well written and it presents the data in a clear manner. The only reservation I have is related to the seemingly low number of animals per treatment.

 Minor comments/suggestions

 Abstract

 ‘Chemoprophylactic prevention’ seems to be a pleonasm (prophylaxis means prevention);

 Is the word ‘veterinary’ before ‘heartworm disease’ needed?

 ‘with or without use of anti-inflammatory treatments’ instead of ‘following use of anti-inflammatory treatments’?

 ‘efficacies achieved using different ….’ instead of ‘efficacies achieved against different ….’?

 ‘evaluation of *D. immitis* development in …’ instead of ‘evaluation of *D. immitis* susceptibility in …’?

 Keywords

 Anthelmintics are antiparasitic drugs, do you need both keywords?

 Research highlights

 What do the authors mean by ‘chronic procedures’? Is it the fact that in dogs heartworm disease commonly runs a chronic course? How did the authors get the ‘67%’ value?

 Introduction

 It seems that some sentences need a little reformulation for their meaning to become clear:

- Humans are at risk of developing abbreviated zoonotic infections causing pulmonary lesions, with increasing reported incidence (Reddy, 2013), frequently confused with tumours (Saha et al., 2022).
- Melarsomine is not registered for use in cats and risks severe adverse events in dogs, requiring complex protracted veterinary clinical case management, exercise restriction and supplementary treatments.

It might be useful to provide a reference for the high efficacy of MLs against the 60 days old larvae.

 What do the authors mean by ‘do not significantly vary up to 5 weeks of infection’? The infections with *D. immitis* GAIII isolate in NSG mice do not vary under what aspects?

 Methods

 Animal infections and methods

 Were the control mice in the Oxfendazole trial treated with the vehicle subcutaneously (there is no mention in the text and on Fig. 1 it is stated ‘Vehicle sc’)?

 Discussion

 ‘we demonstrated 90% efficacy’ instead of ‘we demonstrate 90% efficacy’?

 ‘as an alternative or in combination with ivermectin’ instead of ‘as an alternative or combination with ivermectin’?

 ‘for monthly oral prevention of infection with drug-resistant …’ instead of ‘for monthly oral treatment of drug-resistant ..’?

 ‘previously investigated’ instead of ‘prior investigated’?

 ‘eosinophil-dependent immune response’ instead of ‘eosinophil-dependent immunity’?

 ‘avoid experimental use of dogs and cats’ instead of ‘avoid experimental use’?

Is the work clearly and accurately presented and does it cite the current literature?

Yes

If applicable, is the statistical analysis and its interpretation appropriate?

I cannot comment. A qualified statistician is required.

Are all the source data underlying the results available to ensure full reproducibility?

Yes

Is the study design appropriate and is the work technically sound?

Yes

Are the conclusions drawn adequately supported by the results?

Yes

Are sufficient details of methods and analysis provided to allow replication by others?

Yes

Reviewer Expertise:

Veterinary parasitology

I confirm that I have read this submission and believe that I have an appropriate level of expertise to confirm that it is of an acceptable scientific standard.

# Response

**Published:** 2024-07-29

**Authors:** Jessica Dagley

**Affiliations:** Tropical Disease Biology, Liverpool School of Tropical Medicine, Liverpool, Merseyside, UK

Thank you for your feedback. We appreciate the time it has taken to review this article and we take onboard your comments in the updated version.

 Regarding estimates of reduction in cat and dog use for drug screening, whilst it is impossible to precisely define the percentage reduction with each drug development programme situation and methodology varying, we considered one standard approach, generating murine pharmacokinetic (PK) data combined with an in silico prediction of efficacy based on the relationship between PK and in vitro determined pharmacodynamics (PD) using D. immitis L4 generated in mice. This will reduce numbers of animals required for empirical testing in vivo by selecting only compounds predicted to mediate threshold effects and calibrate doses in vivo to negate equivocal efficacy readouts. In tandem, testing empirical efficacy in the new mouse infection model will further reduce the use of cats or dogs, thereby reducing the risk of adverse reaction occurrence in these highly sentient species. In a scenario where traditionally 10 experimental drug regimens would be run in long-term, 6-month dog studies (72 dogs), with potential welfare consequences, this new approach would cut the number to 24 dogs (67% reduction) replaced by short-term, mild procedures in an estimated 48 mice.
